# Preparation and characterization of the enol of acetamide: 1-aminoethenol, a high-energy prebiotic molecule†

**DOI:** 10.1039/d0sc04906a

**Published:** 2020-10-20

**Authors:** Artur Mardyukov, Felix Keul, Peter R. Schreiner

**Affiliations:** Institute of Organic Chemistry, Justus Liebig University Heinrich-Buff-Ring 17 35392 Giessen Germany prs@uni-giessen.de

## Abstract

Amide tautomers, which constitute the higher-energy amide bond linkage, not only are key for a variety of biological but also prebiotic processes. In this work, we present the gas-phase synthesis of 1-aminoethenol, the higher-energy tautomer of acetamide, that has not been spectroscopically identified to date. The title compound was prepared by flash vacuum pyrolysis of malonamic acid and was characterized employing matrix isolation infrared as well as ultraviolet/visible spectroscopy. Coupled-cluster computations at the AE-CCSD(T)/cc-pVTZ level of theory support the spectroscopic assignments. Upon photolysis at *λ* > 270 nm, the enol rearranges to acetamide as well as ketene and ammonia. As the latter two are even higher in energy, they constitute viable starting materials for formation of the title compound.

## Abstract 2

Amide tautomers, which constitute the higher-energy amide bond linkage, not only are key for a variety of biological but also prebiotic processes.

## Graphical abstract

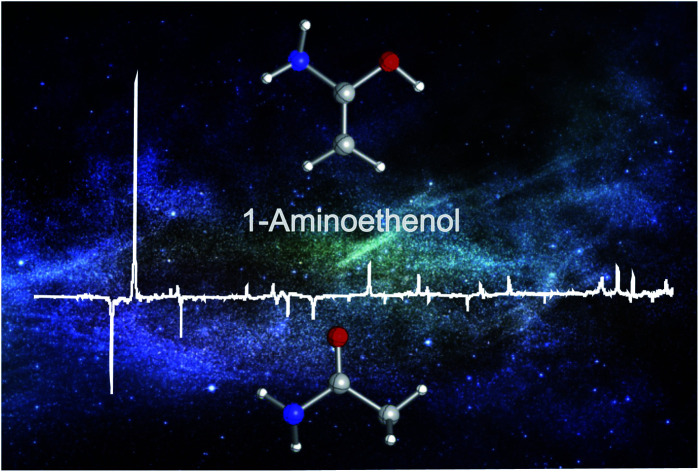

## Introduction

Complex molecular structures typically form from thermodynamically very stable and thus unreactive small molecules that often have to be transformed first into more reactive isomers before they can react. A prime example is ubiquitously available formaldehyde (H_2_CO)^1^ that can only provide a path to forming sugars^2^ through its hydroxymethylene tautomer (H–C̈–OH), a highly reactive carbene, that had long been elusive^3^ but nevertheless had been deemed the “activated form of formaldehyde” for a long time.^4^ Another example is the hydrogenated form of HCN (a molecule abundant in space), namely methanimine (H_2_C=NH) and its tautomer aminocarbene (H–C̈–NH_2_), whose structure has only very recently been revealed.^5^ That is, the higher-energy tautomers of biologically relevant building blocks are likely to play a key role also for the emergence of the molecules relevant to life, but many of these have not been identified and fully characterized.^6–9^

As complex organic molecules including amino acids,^10^ sugars,^11^ purine bases,^12,13^ short peptides,^14^ have been identified in meteorites, prebiotic organic reactions must take place in the interstellar medium through the activation of thermodynamically highly stable small molecules such as CO, CO_2_, H_2_O, CH_4_, HCN but also formaldehyde, simple alcohols, and a variety of carboxylic acids and, particularly relevant to the present study, their amides.^15–17^ The formation of complex organic molecules has been suggested to take place through various scenarios including meteoritic impact.^18–23^ To date, more than 180 molecules^24^ have been identified in the interstellar medium (ISM) through their rotational and vibrational transitions and there is evidence for the existence of larger structures that await their identification.^15,25^ The interstellar presence of (pre)biotic molecules suggests that these may have extraterrestrial origin on earth as well.^26^ A number of these molecules have been detected in the ISM, such as the simplest ‘sugar’ glycolaldehyde^27^ and precursor molecules to the amino acid glycine such as methylamine^28^ and aminoacetonitrile.^29^ Amino acids have been identified in some meteorites^10,30^ but not in interstellar clouds. However, only a few species with a peptide moiety have been detected in interstellar space,^31–33^ with acetamide being the most prominent.^17^ Acetamide has been identified in the environments of Sagittarius B2 (Sgr B2)^17^ and Orion KL,^34^ and in comets^35^ (*e.g.*, 67P/Churyumov–Gerasimenko).^36^ This suggests that aqueous and ambient conditions are not necessarily needed for the production of acetamide.^34^ Acetamide is one of the most abundant complex organic molecules on Sgr B2(N) and has been suggested to be a source of larger peptidic molecules.^34^ The origin of acetamide in the ISM remains unclear, as there is no established formation mechanism that would allow its production in the gas phase.

**Scheme 1:**
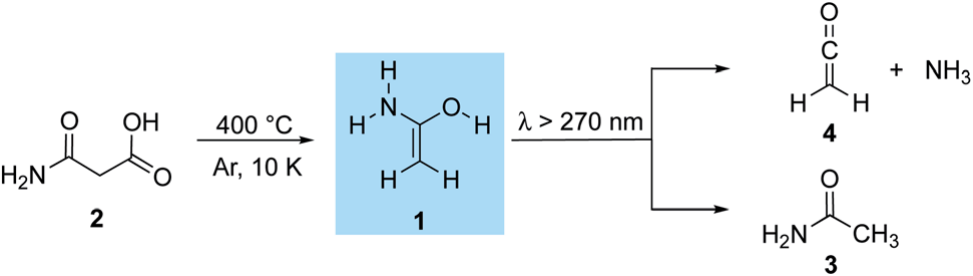
1-Aminoethenol (**1**) synthesized from malonamic acid (**2**) through flash vacuum pyrolysis and trapping in an argon matrix. Subsequent photorearrangement to acetamide (**3**) as well as ketene (**4**) and ammonia.

Foo and colleagues^37^ used computations to propose reaction pathways towards the synthesis of acetamide in the ISM. Eight bimolecular reaction paths were suggested that could lead to the formation of acetamide. 1-Aminoethenol (**1**), the tautomer of acetamide (**3**, Scheme 1), has been suggested to be a key intermediate for the formation of **3** in the ISM,^37^ but has never been observed. To date, only indirect evidence for the experimental preparation of **1**^38,39^ and its enolate^40^ in the gas phase comes from mass spectrometric experiments; there are no spectroscopic data available for **1**.

Amide **3** has been produced in various model ice experiments.^41,42^ Henderson and Gudipati^43^ reported the synthesis of **3** through electron bombardment and/or UV irradiation of H_2_O, NH_3_, and CH_3_OH ices at 5 and 70 K. Belloche *et al.*^33^ suggested the formation of **3** to occur through H-abstraction from formamide followed by methyl radical addition. Garrod *et al.*^44^ proposed that the addition of CH_3_ to HNCO might be responsible for the formation of **3** on grain surfaces. CH_4_–HNCO ice mixtures at 20 K were irradiated with vacuum ultraviolet (VUV) photons to produce the radicals required for the formation of the amides, which then were identified by IR spectroscopy as well as mass spectrometry.^41^ Recently, Haupa *et al.*^45^ found that the reaction of atomic hydrogen with acetamide in solid p-H_2_ at 3.3 K produces the 2-amino-oxoethyl (˙CH_2_CONH_2_) radical, which further dissociates to ketene and ˙NH_2_ upon UV/vis photolysis.

There is growing evidence that enols are building blocks in prebiotic chemistry.^46–48^ Several enols have been postulated to play a key role in the synthesis of carbohydrates in prebiotic cycles.^46^ For instance, 1,2-ethenediol, the enol of glycolaldehyde, has been implied to contribute to the formation of carbohydrates in early Earth environments.^47^ The interstellar detection of the simplest enol (vinyl alcohol, CH_2_=CHOH) in its *syn* and *anti* forms through microwave emissions from Sagittarius B2 was reported in 2001.^49^ Laboratory experiments on plasma discharged of alcohols showed the presence of several enols, suggesting that enols can form from alcohols by high energy (cosmic) irradiation.^50^ Ethenol was thereby generated in the gas phase and characterized by microwave^51,52^ and photoelectron spectroscopy.^53^ Kable *et al.* showed that ethenol forms from acetaldehyde upon UV (310–330 nm) irradiation.^54^ Hawkins and Andrews reported the formation of acetaldehyde, ethylene oxide, ketene, and ethenol as primary products by photolysis of ozone in argon matrices containing ethene.^55^ Nunes *et al.* showed that pyrolysis of isoxazole leads to the formation of (*Z*)-3-hydroxypropenenitrile, which was subsequently photoisomerized by UV-irradiation to its (*E*)-isomer.^56^

Enols of simple amides are rather elusive species due to their high thermodynamic instability and hence high reactivity compared to their corresponding amide isomers, and so far, no clear evidence has been presented regarding their existence.^57^ Only a few examples are known in the literature.^58–61^ Hegarty *et al.*^62^ as well as Wagner *et al.*^63^ suggested the formation of amide enols as intermediates in the hydration of arylketene imines and amination of arylketenes by transient time-resolved spectroscopy. Frey and Rappoport showed the synthesis and NMR spectrum of a conjugated amide enol with bulky substituents (Tip_2_=C(OH)NMe_2_, tip = 2,4,6-triisopropylphenyl) in solution by addition of diethylamine to the corresponding aryl substituted ketene.^64^ The isolation and spectroscopic characterization of unsubstituted enols of amides,^57^ however, has been elusive. To date, only conjugated amide enols were identified using time-resolved spectroscopy.^58,61,63^ Turecek and coworkers^65,66^ described several simple neutral enols that were generated by flash pyrolysis from bicyclo[2.2.1]heptene derivatives and characterized by high-resolution mass spectrometry and ionization energy measurements in the gas phase. Very recently, we reported the preparation of previously elusive 1,1-ethenediol (**7**), the enol of acetic acid, through flash vacuum pyrolysis of malonic acid and subsequent product trapping in argon matrices.^67^

As it is likely that amide enols are potentially detectable interstellar molecules, we present here the generation and spectroscopic characterization of hitherto unreported parent amide enol **1** and its deuterated isotopologue (d_5_-**1**) through flash vacuum pyrolysis of malonamic acid (3-amino-3-oxopropanoic acid, **2**) and d_5_-malonamic acid (d_5_-**2**), respectively. The pyrolysis products were isolated in argon matrices and characterized by IR and UV/vis spectroscopy by matching their spectra with computations at the CCSD(T)/cc-pVTZ level of theory. Apart from being a fundamentally important molecule, our results will also aid the detection of **1** in interstellar space to provide a basis for its involvement in the synthesis of larger biologically relevant molecules.

## Results and discussion

**Fig. 1:**
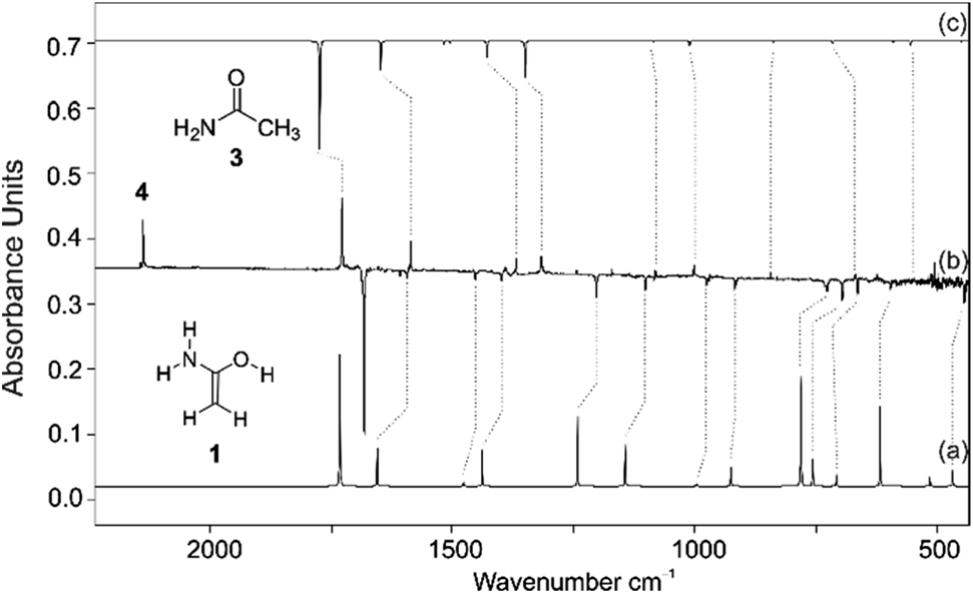
IR spectrum showing the pyrolysis product of **2** with subsequent trapping in an argon matrix at 10 K. (a) IR spectrum of **1** computed at AE-CCSD(T)/cc-pVTZ (unscaled). (b) IR difference spectrum showing the photochemistry of **1** after irradiation with *λ* > 270 nm in argon at 10 K. Downward bands assigned to **1** disappear while upward bands assigned to **3** and **4** appear after 30 min irradiation time. (c) IR spectrum of **3** computed at AE-CCSD(T)/cc-pVTZ (unscaled).

We prepared **1** by thermal extrusion of CO_2_ from **2**, which was evaporated from a storage bulb at 70 °C and passed through a quartz pyrolysis tube, after which the pyrolysis products were co-condensed with a large excess of argon on the surface of the 10 K matrix window. After various attempts, we determined 400 °C as the optimal pyrolysis temperature. Under these conditions, the decarboxylation of **2** is facile as evident by the appearance of the very strong asymmetric stretching vibration of CO_2_ at 2345 cm^−1^. Along with CO_2_, H_2_O, and **3** (*vide infra*), we observed a set of new IR bands that we assign to **1**. The excellent match in the direct comparison of the experimental and the *ab initio* computed [AE-CCSD(T)/cc-pVTZ] IR spectrum confirms this finding. Notably, a strong band at 1682 cm^−1^ is attributed to the C=C stretching mode in **1** (Fig. 1, note that **1** is non-planar due to pyramidalization around nitrogen) that agrees well with previous IR measurements for conjugated (amide)enols. Wagner and colleagues^63^ observed a transition band at 1680 cm^−1^ in the reaction of phenyl ketene with diethylamine, which was attributed to the C=C stretch of the corresponding enol amide. The very prominent bands at 3647, 3510, and 3413 cm^−1^ can be assigned to the OH stretching mode as well as the asymmetric and symmetric NH_2_ stretching modes of **1**, respectively (Fig. S1†). A less intense NH_2_ deformation band mode was found at 1607 cm^−1^ along with bands at 727 and 694 cm^−1^ for the CH_2_ wagging and as well as out of plane rocking modes. With the aid of the computations additional medium and weakly intense bands were attributed to **1** (Table S1†). We also observed other intense bands at 1727, 1586, 1367, and 1313 cm^−1^ in the IR spectrum of the pyrolysis mixture, which were attributed to **3** that forms from **1***via* 1,3[*H*] shift in the gas phase. Structure **3** was identified by comparison with matrix-isolated data of an authentic sample. A very prominent band at 2138 cm^−1^ is assigned to ketene (**4**) (*vide infra*).^68^

The formation of **1** is also supported by isotopic labelling experiments using d_5_-**2** (Fig. S2†). Large isotope red-shifts are found for the OH as well as the NH_2_ asymmetric and symmetric stretching modes of 955, 893, and 928 cm^−1^, in good agreement with the unscaled computed shifts of 1048, 957, and 985 cm^−1^. The shift of the C=C stretch is −51 cm^−1^ (calc. −59 cm^−1^). The scissoring vibration of the CH_2_ group shows an isotope shift of −349 cm^−1^ (calc. −346 cm^−1^). The CH_2_ in-plane and out of plane modes exhibit red shifts of 199 and 150 cm^−1^, again in good agreement with the computed shifts of 202 and 162 cm^−1^, respectively. Overall, the IR frequencies, intensities, and isotopic shifts of two isotopologues (**1** and d_5_-**1**) closely match the computed data (Table S1†), the only exception being the NCC out of plane and NOC in plane deformation modes, for which the computed intensities are higher than those measured; the NCC in plane vibrational frequency could not be ambiguously assigned in the IR spectrum because the low signal-to-noise ratio in this spectral region hampers its identification.

Photolysis of the matrix-isolated pyrolysis products (*λ* > 270 nm) selectively bleached the IR bands of **1** (other species remained unchanged) and simultaneously IR bands matching those of **3** appeared. The formation of **3** from **1** through 1,3[*H*]-migration is also strongly supported by isotopic labelling experiments using d_5_-**1**; irradiation the matrices of d_5_-**1** produces d_5_-**3**. Along with the IR bands of **3**, UV irradiation (*λ* > 270 nm) of matrix isolated **1** resulted in the formation of a strong IR band at 2138 cm^−1^ with an isotopic shift of −27 cm^−1^ (calc. −24 cm^−1^ in d_5_-**4**) that can readily be assigned to the C=O stretching mode of ketene (**4**) as the deamination product of **1**. No other product bands appeared under these conditions.

The photolysis of **3** has been the subject of several studies in the gas phase, solution,^69,70^ in low-temperature matrices,^71^ and computationally.^72^ In argon matrices, VUV irradiation of **3** has been shown to produce HNCO–CH_4_ and CO–CH_3_NH_2_ molecular complexes, and acetimidic acid;^71^ these results could readily be reproduced here. Gas-phase UV-photolysis of **3** results in the formation of CO, ˙CH_3_ and ˙NH_2_ radicals and CH_3_CN through dehydration.^73^ Note that irradiation of **3** (*λ* > 270 nm) does not result in rearrangements or decomposition.

**Fig. 2:**
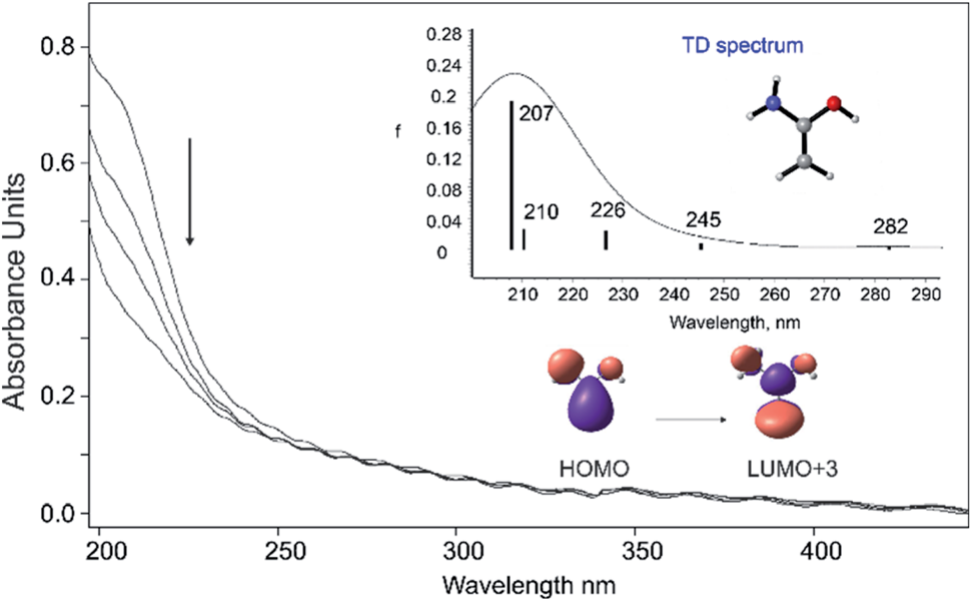
Solid line: UV/vis spectrum of **1** isolated at 10 K in Ar. Dashed lines: the photochemistry of **1** after irradiation at *λ* > 270 nm for 1, 5, and 15 min in argon at 10 K. Inset: computed [TD-B3LYP/6-311++G(2d,p)] electronic transitions for **1**.

The UV/vis spectrum of enol **1** shows a strong absorption band at ∼212 nm, in excellent agreement with the computed value of 207 nm (*f* = 0.190) utilizing time-dependent (TD) density functional theory employing B3LYP/6-311++G(2d,p). Moreover, TD-DFT computations showed weak transitions at 226 nm (*f* = 0.026), 245 nm (*f* = 0.008), and 282 nm (*f* = 0.003). Unfortunately, due to the low extinction coefficients, the UV/vis transitions of **1** much above 212 nm could not be detected. The short-wavelength absorption at ∼212 nm originates from a HOMO–LUMO+3 excitation, which corresponds to a π–π* transition (Fig. 2). In accordance with the IR experiments, irradiation of the matrix with wavelength *λ* > 270 nm leads to complete disappearance of the observed UV band. The computed bands of **3** and **4** in the region at ∼210–215 nm display low extinction coefficients and therefore transitions of **3** and **4** could not be detected (Fig. S3†).

We also computed the potential energy hypersurface surrounding **1**. According to our CCSD(T)/cc-pVTZ computations, **1** exists in two distinct conformations: *anti*-**1** where the OH group has an *anti*-orientation to the NH_2_ group, and *syn*-**1** with a *syn*-orientation of the OH group; *anti*-**1** is 0.9 kcal mol^−1^ (Δ*H*_0_) more stable than *syn*-**1**. The activation enthalpy for the *anti*-**1** → *syn*-**1** conformational isomerization is 3.9 kcal mol^−1^. The effect of nitrogen substitution on the electronic structure of **1** relative to ethylene and other enols can be estimated from the HOMO–LUMO energy differences (Fig. S4†). Substitution of one OH group with NH_2_ leads to destabilization of the HOMO energy, resulting in a smaller HOMO–LUMO gap in **1** (−0.195 eV) compared to 1,1-ethenediol (−0.207 eV). This indicates that **1** is more nucleophilic than 1,1-ethenediol but also that it can be oxidized more readily. Nitrogen as a superior π–donor leads to even larger destabilization of the HOMO with Δ*E*(*E*_HOMO_ − *E*_LUMO_) value of −0.190 eV in 1,1-diaminoethylene (Fig. S4†). The C=C bond of 1.337 Å in **1** is slightly shorter than that in 1,1-ethenediol (1.343 Å, Fig. S5†).^67^ This is also evident from the experimentally observed C=C stretching vibration of **1** that is red-shifted by 30 cm^−1^ in comparison to the C=C stretching vibration of 1712 cm^−1^ in the diol.^67^ By comparison of the experimentally observed IR spectrum with the computed spectra of *anti*-**1** and *syn*-**1**, we conclude that only conformer *anti*-**1** is present in the argon matrix. Indeed, the *syn*-**1** to *anti*-**1** conformational transformation is associated with a very fast computed quantum mechanical tunnelling (QMT) half-life of 2.72 × 10^−8^ s (for additional information please see the ESI†).

**Fig. 3:**
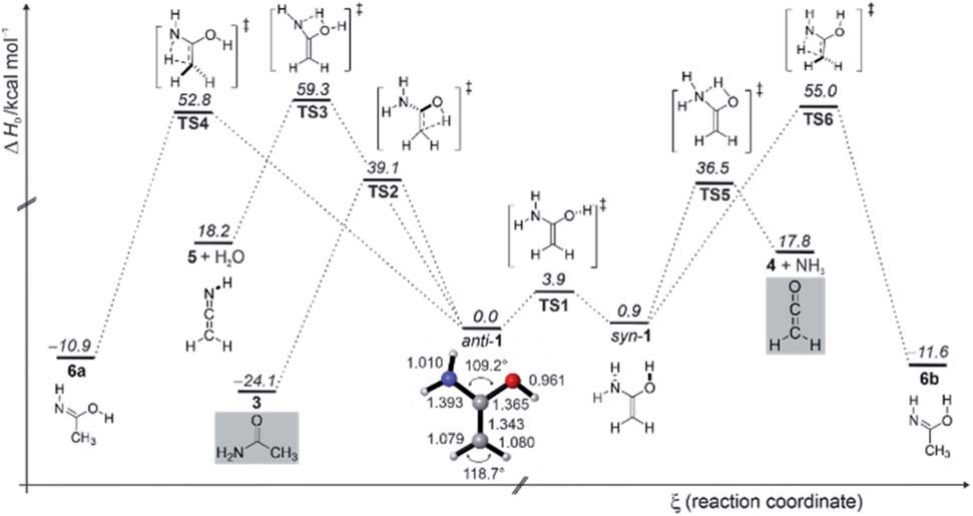
Potential energy profile (Δ*H*_0_) in kcal mol^−1^ of the reactions of enol **1** at AE-CCSD(T)/cc-pVTZ + ZPVE at 0 K.

The enol to amide (**1** → **3**) interconversion proceeds through non-planar transition structure (**TS2**) and requires activation of 39.1 kcal mol^−1^ (Fig. 3), which is similar to [1,3]*H*-shift in 1,1-ethenediol to acetic acid.^67^ Generally speaking, such [1,3]*H*-shifts are highly unfavourable because they involve 4-electron/4-center transition structures. The related [1,3]*H*-shift from *syn*-**1** to acetimidic acid (**6**) is characterized by an even higher barrier (**TS5**) of 54.1 kcal mol^−1^. The formation of ketenimine **8** and water from *anti*-**1** is associated with a similarly high barrier (**TS4**) of 59.7 kcal mol^−1^; **5** has been detected in the star-forming region Sagittarius B2(N).^74^ We did not observe **5** and **6** in our FVP experiments, in accordance with our computations that render these two reactions rather unlikely at FVP temperature of ∼400 °C due to the excessively high activation barriers. We do observe the characteristic peaks of **3** and **4** directly after FVP, suggesting that the energy in the pyrolysis zone is sufficiently high for **1** to undergo a [1,3]*H*-shift to **3** (Δ*H*_0_^‡^ = 39.1 kcal mol^−1^) and decomposition to produce **4** and ammonia (Δ*H*_0_^‡^ = 35.6 kcal mol^−1^). Formation of acetimidic acid (**6**, two conformers are shown in Fig. 3) from **1** is exothermic by −11 to −12 kcal mol^−1^ but requires 55 kcal mol^−1^ activation. In contrast to **1**, **4**, and **6** have been synthesized and characterized in argon matrices by vacuum ultraviolet irradiation of **3** by Duvernay *et al.*^71^

Apart from fast C–O bond rotation, **1** does not display QMT reactivity because the barriers are generally too high and too wide.^75^ This is evident from the fact that when keeping matrices containing **1** in the dark at 10 K the IR bands of **1** remain unchanged over the course of six days.

The spectroscopic proof for the existence of **1** is important in the context of the abiotic formation of heteroatomic organic molecules that are relevant for the formation of biologically relevant building blocks. Our studies can aid the identification of **1** in interstellar media. As both ketene and ammonia have been detected in interstellar clouds,^76,77^ the discovery of **1** is only a matter of time because this bimolecular reaction is *ca.* −18 kcal mol^−1^ exothermic. The barrier of this amination is too high to occur under interstellar conditions, it may well be significantly lowered through NH_3_ and/or H_2_O catalysis on ices or dust grains, as evident from numerous studies^78,79^ showing that the activation energy for the addition of NH_3_ to ketene can be significantly lowered through a second ammonia molecule that acts as a bifunctional catalyst.^78^

## Author contributions

A. M. and P. R. S. conceived the idea. A. M. carried out all experimental and computational studies. F. K. synthesized the starting material. A. M. and P. R. S. analysed and discussed all data. A. M. and P. R. S. co-wrote the manuscript.

## Conflicts of interest

No competing financial interests have been declared.

## Supplementary Material

SC-011-D0SC04906A-s001
