# Supplementary material for: Preparation and characterization of the enol of acetamide: 1-aminoethenol, a high-energy prebiotic molecule
Source: Chem Sci. 2020 Oct 20;11(45):12358–63. doi: 10.1039/d0sc04906a (PMC8162875; doi:10.1039/d0sc04906a)
Supplement: SC-011-D0SC04906A-s001 [file SC-011-D0SC04906A-s001.pdf]

Electronic Supplementary Material (ESI) for Chemical Science.
This journal is © The Royal Society of Chemistry 2020

Preparation and Characterization of the Enol of Acetamide: 1-Aminoethenol, a High-Energy Prebiotic Molecule

Artur Mardyukov,[a] Felix Keul,[a] and Peter R. Schreiner[a]*

Table of Contents

Experimental Procedures S2
Figure S1 ..... S3
Figure S2 ..... S4
Figure S3 ..... S4
Figure S4 ..... S5
Figure S5 ..... S5
Table S1. ..... S6
References S6
AE-CCSD(T)/cc-pVTZ optimized structures S8

Experimental Procedures

Matrix Apparatus Design. For the matrix isolation studies, we used an APD Cryogenics HC-2 cryostat with a closed-cycle refrigerator system, equipped with an inner CsI window for IR measurements. Spectra were recorded with a Bruker IFS 55 FT-IR spectrometer with a spectral range of 4500−400 cm^{-1} and a resolution of 0.7 cm^{-1} and UV/Vis spectra were recorded with a JASCO V-670 spectrophotometer (UV/Vis band width of 1.0 nm) equipped with an inner sapphire window. A high-pressure mercury lamp (HBO 200, Osram) with a cut-off filter was used for irradiation.

For the combination of high-vacuum flash pyrolysis with matrix isolation, we employed a small, home-built, water-cooled oven, which was directly connected to the vacuum shroud of the cryostat. The pyrolysis zone consisted of an empty quartz tube with an inner diameter of 8 mm, which was resistively heated over a length of 50 mm by a coaxial wire. The temperature was monitored with a NiCr−Ni thermocouple. Malonamic acid **2** (Sigma-Aldrich) was evaporated (**2**: 70 °C) from a storage bulb into the quartz pyrolysis tube. At a distance of approximately 50 mm, all pyrolysis products were co-condensed with a large excess of argon (typically 60−120 mbar from a 2000 mL storage bulb: circa 50-60 mbar per hour) on the surface of the matrix window at 10 K. Several experiments with pyrolysis temperatures ranging from 200 to 700 °C were performed in order to determine the optimal pyrolysis conditions.

Synthesis of 2. 122 mg (3.05 mmol, 1.00 equiv.) NaOH was dissolved in 10 mL H_2O and 400 mg (3.05 mmol, 1.00 equiv.) ethyl malonate monoamide was added and the resulting mixture was stirred for 3 h under reflux. The solution was cooled to room temperature and acidified with 1 M HCl solution to pH 4. The lyophilization of the mixture at –90 °C gave **2** (314 mg, >99%, quantitative). ^{1}H-NMR (400 MHz, D_2O): δ/ppm 3.29 (s, CH_2), 3.28 (t, J = 2.3 Hz, CHD); ^{13}C-NMR (101 MHz, D_2O): δ/ppm 174.00, 173.38, 43.69, 43.65, 43.45, 43.25. Note: due to the acidity of the CH_2 group we observed proton exchange with D_2O. Therefore we observed four signals for this group.

Synthesis of *d*5-2. 100 mg of **2** was dissolved in 3 mL D_2O. After stirring overnight at room temperature, the solvent was removed under reduced pressure. This procedure was repeated 3-5 times. ^{13}C-NMR (101 MHz, D_2O): δ/ppm 174.4, 174.3, 43.6.

Computations. All coupled cluster computations were carried out with the CFOUR[1] program package. In general, all electron coupled cluster level of theory[2] including single, double, and perturbatively included triple excitations [AE-CCSD(T)] utilizing the Dunning correlation consistent split valence basis set cc-pVTZ[3] was employed for geometry optimizations and frequency computations. For B3LYP/6-311++G(2d,2p)[4] computations we used the Gaussian16[5] program package. The quantum mechanical tunneling computations were carried out using the *Polyrate* computer code that is available at no cost at http://truhlar.chem.umn.edu/content/software.[6]

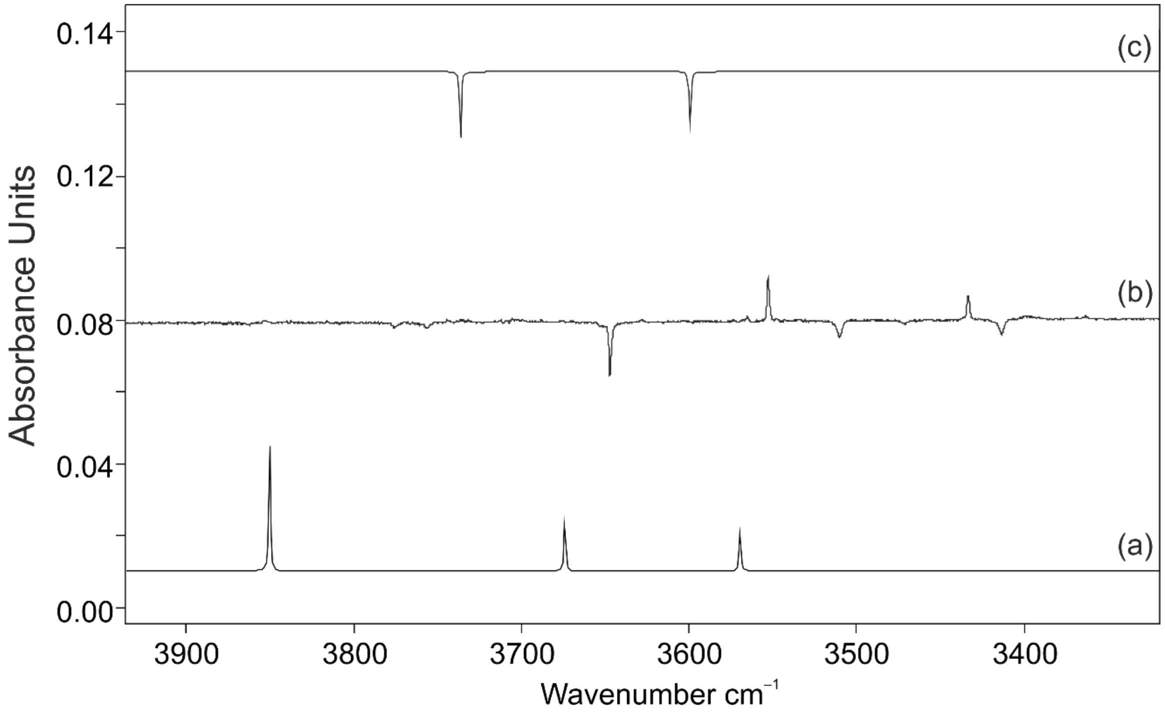

Figure S1. IR spectra showing the pyrolysis product of **2** with subsequent trapping in an argon matrix at 10 K. (a) IR spectrum of **1** computed at AE-CCSD(T)/cc-pVTZ (unscaled). (b) IR difference spectra showing the photochemistry of **1** after irradiation with λ > 270 nm in argon at 10 K. Downward bands assigned to **1** disappear while upward bands assigned to **3** and **4** appear after 30 min irradiation time. (c) IR spectrum of **3** computed at AE-CCSD(T)/cc-pVTZ (unscaled).

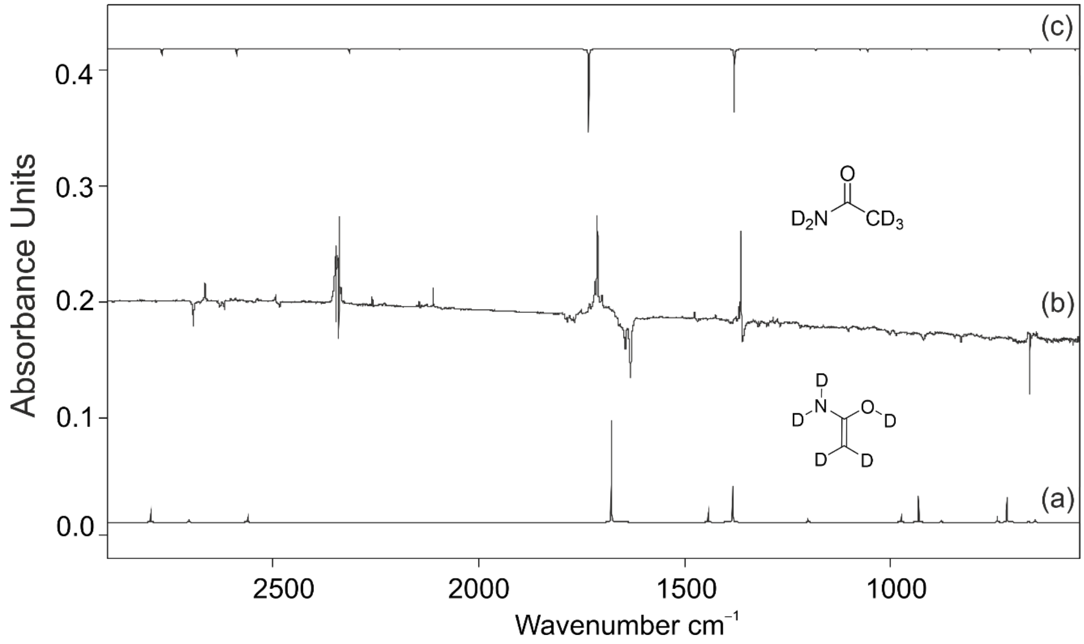

Figure S2. IR spectra showing the pyrolysis product of d_5-**2** with subsequent trapping in an argon matrix at 10 K. (a) IR spectrum of d_5-**1** computed at AE-CCSD(T)/cc-pVTZ (unscaled). (b) IR difference spectra showing the photochemistry of d_5-**1** after irradiation with λ > 270 nm in argon at 10 K. Downward bands assigned to d_5-**1** disappear while upward bands assigned to d_5-**3** and d_2-**4** appear after 30 min irradiation time. (c) IR spectrum of d_5-**3** computed at AE-CCSD(T)/cc-pVTZ (unscaled).

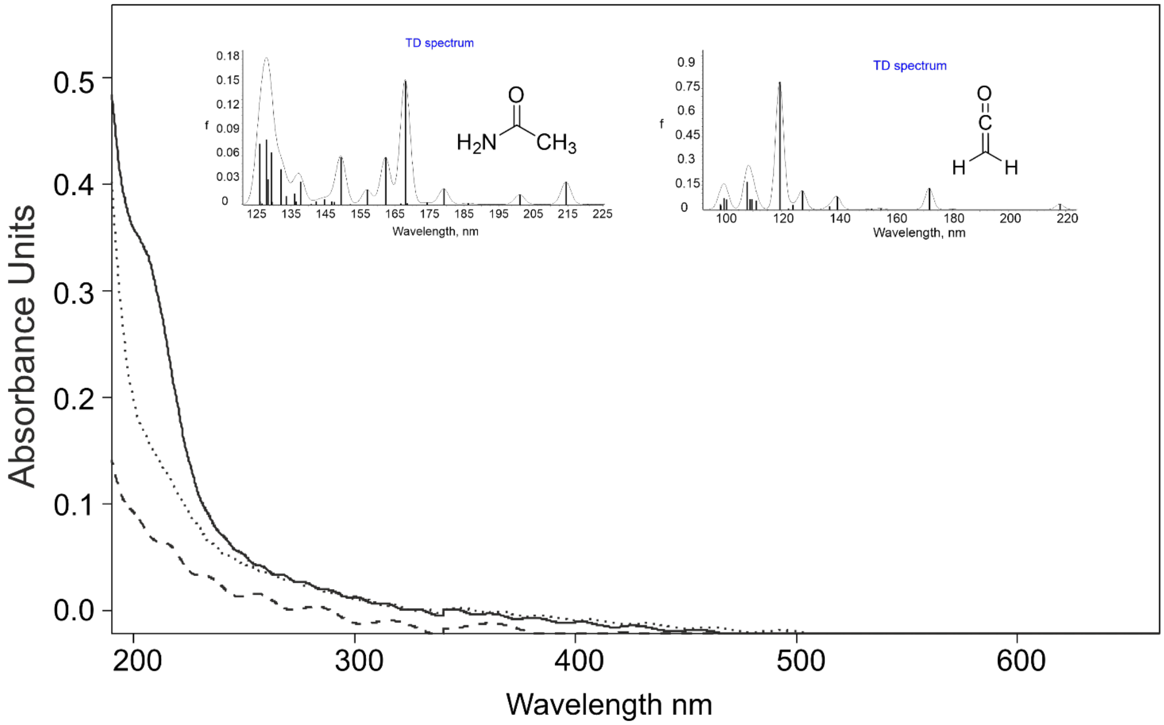

Figure S3. Solid line: UV/Vis spectrum of **1** isolated at 10 K in Ar. Dot line: the photochemistry of **1** after irradiation at λ > 270 nm for 15 min in argon at 10 K. Dashed line: UV/Vis spectrum of **3** isolated at 10 K in Ar. Inset: computed [TD-B3LYP/6-311++G(2d,2p)] electronic transitions for **3** and **4**.

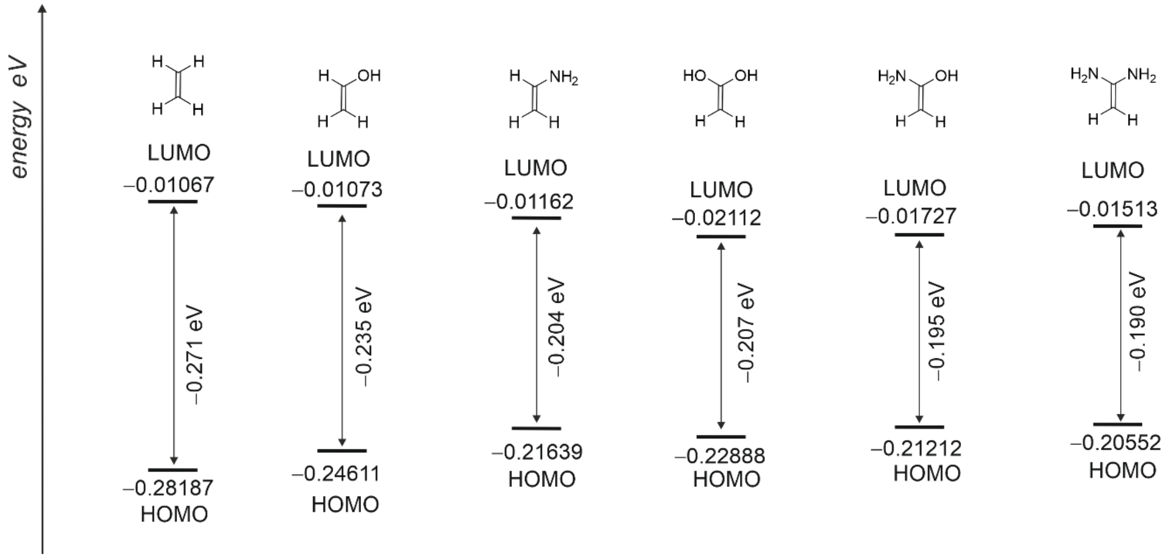

Figure S4. HOMO – LUMO energies of ethylene, and variety of enols computed at the B3LYP/6-311++G(2d,2p) level of theory.

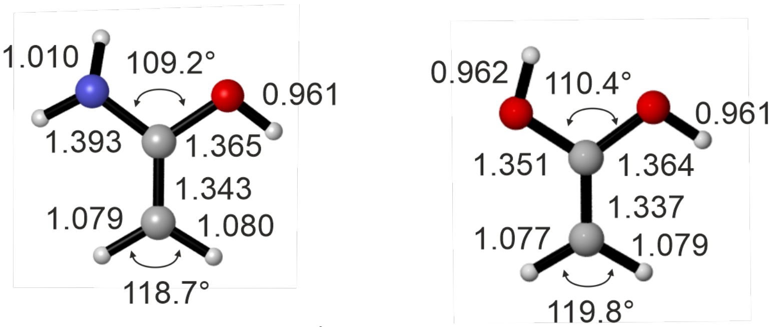

Figure S5. Bond lengths (Å) and angles of **1** and **7** at the CCSD(T)/cc-pVTZ level of theory.

Table S1. Experimental (Ar matrix, 10 K) and computed IR frequencies of **1** and *d*$_5$-**1**, peak positions in cm^{-1}, computed infrared intensities (km mol^{-1}) in parentheses.

Mode	**1** Computed[a]	**1** Ar, 10 K[b]	*d*$_5$-**1** Computed[a]	*d*$_5$-**1** Ar, 10 K[b]	Assignment
21	3849 (48.1)	3647 (s)	2801 (29.9)	2692 (s)	OH str.
20	3652 (15.2)	3510 (s)	2695 (10.1)	2617 (s)	NH_2 asym str.
19	3550 (14.5)	3413 (s)	2565 (14.6)	2482 (m)	NH_2 sym. str.
18	3229 (7.2)	3132 (w)	2407 (2.3)		CH_2 asym. str.
17	3161 (0.7)	-	2310 (11.7)	2280	CH_2 sym. str.
16	1732 (279.2)	1682 (s)	1673 (270.7)	1631 (s)	C=C str.
15	1655 (53.5)	1607 (w)	1217 (9)	1197 (w)	NH_2 bending
14	1477 (6.3)	1452 (m)	1131 (16.9)	1103 (m)	CH_2 scissoring
13	1438 (47.3)	1398 (m)	1394 (155.8)	1360 (s)	C-O str.
12	1242 (97.6)	1202 (s)	958 (62.5)	920 (m)	OH def.
11	1143 (78.5)	1001 (m)	914 (13.5)	890 (w)	COH in pl. def.
10	997 (4.9)	974 (m)	795 (5.6)	775 (w)	CCH in pl. def.
9	925 (26.8)	916 (m)	840 (13.4)	829 (m)	C-O str. / C-N str.
8	780 (223)	730 (m)	716 (65.9)	692 (m)	NCC out of pl. def.
7	756 (42.6)	727 (m)	594 (28.1)	577 (w)	CH_2 wagging
6	707 (12.5)	694 (m)	552 (39.8)	552 (m)	CH_2 twisting
5	617(115.7)	594 (m)	473 (82.9)	465 (m)	C-O wagging
4	513 (10)	500 (w)	456 (7.3)	449 (w)	NOC in pl. def.
3	467 (21.5)	441 (m)	385 (13.9)	-	NCC in pl. def.
2	424 (70.6)	411 (m)	308 (42)	-	O-H wagging
1	276 (44.1)	-	200 (22.3)	-	NH_2 twisting

[a]AE-CCSD(T)/cc-pVTZ, harmonic approximation, unscaled frequencies, intensities (in parentheses) in km mol^{-1}. [b] Experiment: argon matrix, 10 K.; approximate relative intensities (w: weak, m: medium, s: strong).

References

[1] CFOUR, **2010**, http://www.cfour.de. J.F. Stanton, J. Gauss, L. Cheng, M.E. Harding, D.A. Matthews, P.G. Szalay with contributions from A.A. Auer, R.J. Bartlett, U. Benedikt, C. Berger, D.E. Bernholdt, S. Blaschke, Y.J. Bomble, S. Burger, O. Christiansen, D. Datta, F. Engel, R. Faber, J. Greiner, M. Heckert, O. Heun, M. Hilgenberg, C. Huber, T.-C. Jagau, D. Jonsson, J. Jusélius, T. Kirsch, K. Klein, G.M. Kopper, W.J. Lauderdale, F. Lipparini, T. Metzroth, L.A. Mück, T. Nottoli, D.P. O'Neill, D.R. Price, E. Prochnow, C. Puzzarini, K. Ruud, F. Schiffmann, W. Schwalbach, C. Simmons, S. Stopkowicz, A. Tajti, J. Vázquez, F. Wang, J.D. Watts and the integral packages *MOLECULE* (J. Almlöf and P.R. Taylor), *PROPS* (P.R. Taylor), *ABACUS* (T. Helgaker, H.J. Aa. Jensen, P. Jørgensen, and J. Olsen), and ECP routines by A. V. Mitin and C. van Wüllen.

[2] a) J. Čížek, *J. Chem. Phys.* **1966**, *45*, 4256-4266; b) R. J. Bartlett, J. D. Watts, S. A. Kucharski, J. Noga, *Chem. Phys. Lett.* **1990**, *165*, 513-522; c) K. Raghavachari, *Annu. Rev. Phys. Chem.* **1991**, *42*, 615-642; d) J. F. Stanton, *Chem. Phys. Lett.* **1997**, *281*, 130-134.

[3] T. H. Dunning, Jr., *J. Chem. Phys.* **1989**, *90*, 1007-1023.

[4] a) A. D. Becke, *Phys. Rev. A* **1988**, *38*, 3098-3100; b) A. D. Becke, J. *Chem. Phys.* **1993**, *98*, 5648-5652; c) C. Lee, W. Yang, R. G. Parr, *Phys. Rev. B* **1988**, *37*, 785-789. d) S. H. Vosko, L. Wilk and M. Nusair, *Can. J. Phys.*, 1980, **58**, 1200. e) R. C. Binning Jr. and L. A. Curtiss, *J. Comput. Chem.*, **11** (1990) 1206-16

[5] Gaussian 16, Revision C.01, M. J. Frisch, G. W. Trucks, H. B. Schlegel, G. E. Scuseria, M. A. Robb, J. R. Cheeseman, G. Scalmani, V. Barone, G. A. Petersson, H. Nakatsuji, X. Li, M. Caricato, A. V. Marenich, J. Bloino, B. G. Janesko, R. Gomperts, B. Mennucci, H. P. Hratchian, J. V. Ortiz, A. F. Izmaylov, J. L. Sonnenberg, D. Williams-Young, F. Ding, F. Lipparini, F. Egidi, J. Goings, B. Peng, A. Petrone, T. Henderson, D. Ranasinghe, V. G. Zakrzewski, J. Gao, N. Rega, G. Zheng, W. Liang, M. Hada, M. Ehara, K. Toyota, R. Fukuda, J. Hasegawa, M. Ishida, T. Nakajima, Y. Honda, O. Kitao, H. Nakai, T. Vreven, K. Throssell, J. A. Montgomery, Jr., J. E. Peralta, F. Ogliaro, M. J. Bearpark, J. J. Heyd, E. N. Brothers, K. N. Kudin, V. N. Staroverov, T. A. Keith, R. Kobayashi, J. Normand, K. Raghavachari, A. P. Rendell, J. C. Burant, S. S.

Iyengar, J. Tomasi, M. Cossi, J. M. Millam, M. Klene, C. Adamo, R. Cammi, J. W. Ochterski, R. L. Martin, K. Morokuma, O. Farkas, J. B. Foresman, and D. J. Fox, Gaussian, Inc., Wallingford CT, 2016.

[6] J. Zheng, J. L. Bao, R. Meana-Pañeda, S. Zhang, B. J. Lynch, J. C. Corchado, Y.-Y. Chuang, P. L. Fast, W.-P. Hu, Y.-P. Liu, G. C. Lynch, K. A. Nguyen, C. F. Jackels, A. Fernandez Ramos, B. A. Ellingson, V. S. Melissas, J. Villà, I. Rossi, E. L. Coitiño, J. Pu, T. V. Albu, A. Ratkiewicz, R. Steckler, B. C. Garrett, A. D. Isaacson, and D. G. Truhlar, *Polyrate* version 2017-C (University of Minnesota, Minneapolis, MN, 2017).

AE-CCSD(T)/cc-pVTZ optimized structures (distances in bohr), electronic energies (in hartree) and zero-point vibrational energies (ZPVE).

anti-**1**: 1-Aminoethenol
0 1

6	1.316987603	-0.601746151	-0.010157044
1	1.444004235	-1.673309971	-0.035372377
1	2.196160361	0.024119650	0.044987051
6	0.087370942	-0.060434280	-0.003329160
8	-0.166528805	1.281669446	0.003711935
7	-1.118289881	-0.757196942	0.060182572
1	0.680211601	1.733932295	-0.052757151
1	-1.841807929	-0.298567106	-0.475518819
1	-1.019180633	-1.721970545	-0.215869630

E = -208.8510944
ZPVE= 0.07427201

Syn-**1**: 1-Aminoethenol
0 1

6	1.384870625	-0.427470879	-0.002805692
1	1.641772193	-1.471522282	-0.102299318
1	2.168695073	0.307465497	0.094196080
6	0.103117945	-0.042390580	-0.005854918
8	-0.241709403	1.281182592	-0.025845562
7	-1.023452755	-0.887195504	0.052763560
1	-1.081849338	1.350528027	0.441636166
1	-1.635188445	-0.737561598	-0.741590076
1	-0.754339692	-1.860642110	0.088249985

E = -208.8498955
ZPVE = 0.074551528

3: acetamide
0 1

6	0.046143357	-0.085710347	0.000000000
8	0.432840695	-1.247545668	0.000000000
7	0.909976685	0.982917074	0.000000000
6	-1.437338401	0.284383325	0.000000000
1	1.906622765	0.792166917	0.000000000
1	0.589211339	1.941926529	0.000000000
1	-1.910384173	-0.165190939	0.890961833
1	-1.623375377	1.373184295	0.000000000
1	-1.910384173	-0.165190939	-0.890961833

E = -208.8891525
ZPVE = 0.073922852

4: ketene
0 1

8	0.000000000	0.000000000	-1.187699589
6	0.000000000	0.000000000	-0.020723401
6	0.000000000	0.000000000	1.297853416
1	-0.941865752	0.000000000	1.821543060
1	0.941865752	0.000000000	1.821543060

E = -152.3583735
ZPVE = 0.031634735

5: ketenimine

0 1

1	0.811550531	-1.735064771	0.000000000
7	-0.080805138	-1.243042454	0.000000000
6	0.025089309	-0.014741079	0.000000000
6	0.000939454	1.301809217	0.000000000
1	0.000632166	1.840716104	-0.935570967
1	0.000632166	1.840716104	0.935570967

E = -132.4808658
ZPVE = 0.04381128

6: acetimidic acid
0 1

1	-1.695067798	-0.992424225	0.000000000
7	-0.693935035	-1.194139888	0.000000000
6	-0.049031468	-0.101727129	0.000000000
8	-0.682044724	1.116191481	0.000000000
1	-0.018982906	1.813018591	0.000000000
6	1.451983751	-0.052413472	0.000000000
1	1.847824119	-1.063963639	0.000000000
1	1.813917589	0.477862021	0.884777248
1	1.813917589	0.477862021	-0.884777248

E = -208.8684676
ZPVE = 0.074219899

6a: *syn* acetimidic acid
0 1

1	-1.705948656	-1.029232329	0.000000000
7	-0.697683653	-1.196162033	0.000000000
6	-0.050357068	-0.100165289	0.000000000
6	1.446845599	-0.052401936	0.000000000
8	-0.575369046	1.163662824	0.000000000
1	-1.536608465	1.088650647	0.000000000
1	1.842098241	-1.064077640	0.000000000
1	1.799047270	0.486485943	0.880974094
1	1.799047270	0.486485943	-0.880974094

E = -208.8696503
ZPVE = 0.074322368

Ammonia
0 1

7	0.070567151	0.000000000	0.000000000
1	-0.326828241	0.466495135	-0.807993275
1	-0.326828241	-0.932990270	0.000000000
1	-0.326828241	0.466495135	0.807993275

E = -56.47319724
ZPVE = 0.034550228

Water
0 1

8	0.000000000	0.000000000	0.066414153
1	0.000000000	-0.753879329	-0.527020397
1	0.000000000	0.753879329	-0.527020397

E = -76.33221652
ZPVE = 0.02153226

7: 1,1-Ethenediol

0 1
1 -0.018795953 1.876089259 0.000000000
8 -0.625977817 1.130191040 0.000000000
6 0.113833334 -0.016309539 0.000000000
8 -0.694721943 -1.098785301 0.000000000
1 -1.600631789 -0.772277128 0.000000000
6 1.448596922 -0.096228935 0.000000000
1 1.926045777 -1.062233251 0.000000000
1 2.050256108 0.799965571 0.000000000
E = -228.708374080697

TS1
0 1
6 1.278209034 -0.673629139 -0.014949945
1 1.353620903 -1.751835885 -0.010462993
1 2.176444895 -0.077793675 0.014684372
6 0.083316587 -0.068829667 -0.009242255
8 -0.024359701 1.307782903 0.059804729
7 -1.159806614 -0.712044830 0.059225597
1 -0.199332211 1.637097716 -0.826908192
1 -1.899702450 -0.173106663 -0.365114738
1 -1.141117973 -1.656095126 -0.296191656
E = -208.8436818
ZPVE = 0.073133701

TS2
0 1
6 1.130385221 -0.877580877 -0.037290036
1 1.057461919 -1.861699400 -0.492555458
1 1.645437719 -0.883347268 0.922257105
6 -0.042171056 -0.040567442 0.021119496
8 0.327945238 1.200829376 -0.002327692
7 -1.337193040 -0.362071101 0.003516303
1 -2.030981520 0.354635505 -0.118085781
1 -1.624617552 -1.309633002 0.155952975
1 1.370219352 0.604987991 -0.286943575
E = -208.7831629
ZPVE = 0.068711139

TS3
0 1
6 1.530098447 0.062739498 -0.007857107
1 2.217192389 -0.759026985 -0.140958108
1 1.906235921 1.053902101 0.185889813
6 0.211611118 -0.196189486 0.011281768
8 -0.731773563 1.057619534 0.057277325
7 -0.761432863 -1.092642040 -0.039174891
1 -0.688626477 1.490166737 -0.809139092
1 -1.442670957 0.211760897 0.045186392
1 -0.536975930 -2.011474909 0.313521158
E = -208.7499644
ZPVE = 0.067686932

TS4
0 1
6 -1.099178702 0.918924532 0.019222035

1	-1.536946921	1.034686712	-0.970545355
1	-1.003422608	1.857689179	0.561470317
6	0.029249426	0.013810590	-0.005431793
8	1.346431882	0.264261979	0.003464296
7	-0.441646802	-1.212103291	0.035376351
1	1.457589845	1.206199922	-0.160502481
1	0.114329554	-1.992461723	-0.313008814
1	-1.524540932	-0.564673072	0.171875971

E = -208.7609226
ZPVE = 0.068361981

TS5
0 1

6	1.505553682	-0.222206771	0.000000000
1	1.825754898	-1.252698240	0.000000000
1	2.242264532	0.566254116	0.000000000
6	0.199013689	0.090987419	0.000000000
8	-0.535902160	1.163247901	0.000000000
7	-0.902539008	-0.955838117	0.000000000
1	-1.437370829	0.131999714	0.000000000
1	-0.940645459	-1.531993986	-0.834627538
1	-0.940645459	-1.531993986	0.834627538

E = -208.7882989
ZPVE = 0.069797975

TS6
0 1

6	1.109558710	0.893944699	-0.023119262
1	1.646503651	0.907286691	0.922043037
1	1.026978616	1.864632715	-0.501571633
6	-0.025618952	0.025064687	0.016781174
8	-1.314210994	0.393549289	0.022178872
7	0.412946154	-1.227777765	-0.048209175
1	-1.855434635	-0.364512127	-0.229295428
1	-0.113410447	-1.968088851	0.421446223
1	1.508941501	-0.568544200	-0.219315262

E = -208.757561
ZPVE = 0.068387479
